# Editorial: Therapies and influences in urothelial carcinoma

**DOI:** 10.3389/fonc.2022.1126494

**Published:** 2022-12-29

**Authors:** G. Ozgun, S. Isharwal, B. J. Eigl

**Affiliations:** ^1^ Department of Medical Oncology, BC Cancer Vancouver Center, Vancouver, BC, Canada; ^2^ University of Virginia, Charlottesville, VA, United States

**Keywords:** upper tract urothelial carcinoma, urothelial carcinoma (UC), immunotherapy, molecular targets, radiomics, ASA score, telomere, diabetes

Urothelial carcinoma (UC) is one of the most common cancers worldwide, with more than 570,000 new cases per year. Despite recent therapeutic advances, long-term survival rates for metastatic UC remain poor. There is an urgent need not just for better therapies but also for clinical indicators, biomarkers, and treatment approaches for UC. This Research Topic encompasses a selection of research papers in UC, including molecular research, new therapeutic approaches, treatment guidance, and prediction of treatment response that may influence future directions.

Nelson et al. set the stage with a thorough review of the many different approaches and molecular targets currently being exploited or explored for anti-UC therapeutics. There are already at least 4 different classes of therapies (cytotoxic chemotherapy, immunotherapy, small molecule inhibitors, antibody-drug conjugates) that are FDA approved for the treatment of advanced UC. We are reminded of the recent and rapid explosion of our understanding of UC as a disease that can be sub-classified according to differential RNA expression and further stratified through genomic and biomarker analyses such that targeted molecular therapies will play a larger role along with established therapies.

Although immunotherapies have opened a new era in the treatment of bladder cancer, only a limited number of patients respond to treatment. Currently available markers such as tissue PD-L1 staining fails as predictive biomarkers of response, and a clinically useful marker is desperately needed. Using mass and flow cytometry techniques, Lavoie et al. evaluated the circulating immune compartment of patients exposed to PD-1 inhibitors. They discovered marked differences and dynamic changes in the frequency of specific immune cells after treatment. The main finding was that higher frequencies of naïve CD4+ T cells, lower frequencies of CD161+ Th17 cells, and CCR4+ Th2 cells were found in responders. Overall, a less naive and more activated T-cell phenotype was found in non-responder patients. They also showed a clonal expansion of γ/δ-T cells in a responder, which might serve as a new marker. While this is a small proof-of-concept study, if validated, this approach could allow for non-invasive, serial sampling of the circulating immune compartment to aid proactive monitoring of response to immunotherapies.

Telomere maintenance is one of the hallmarks of cancer, and telomerase-specific mutations are among the most common in UC (1). Telomerase consists of reverse transcriptase (TERT) and telomerase RNA portions. Lack of telomerase activity entailing cellular senescence is a normal process in aging non-malignant cells. Conversely, through different mechanisms, most malignant cells preserve telomere length. There is growing evidence that TERT promoter mutation causing genomic instability is a critical step in the tumorigenesis of UC, which is detected in 73% of bladder UC and 53% of upper tract UC patients (2). In addition, TERT promoter mutation assessed with urine liquid biopsy correlates with mutation rates in UC tissues, and C228T mutation, along with other TERT promoter mutations, has been shown to be detectable up to 10 years before the development of UC in urine liquid biopsies of patients with invisible tumors (3). Hayashi et al. reported that TERT C228T mutation in urinary cfDNA was associated with bladder tumor recurrence after surgery for non-muscle invasive bladder cancer or upper tract UC (UTUC). Hence, detecting TERT C228T mutation in urine samples might be utilized as a prognostic factor and a diagnostic biomarker.

Several other articles in this Research Topic focus on clinical factors relevant to UC management. For the assessment of surgical risk, the American Society of Anesthesiologists-physical status (ASA-PS) classification presents a 6-point comorbidity scale. While ASA scores (≥2) were independently associated with poorer overall survival, cancer-specific survival, and metastasis-free survival, no significant differences in intravesical recurrence-free survival were observed among the different ASA scores (Yuan et al.). This simple score may represent a useful tool for clinical evaluation of patients undergoing radical nephroureterectomies. On another note, in predicting the response of patients with muscle invasive bladder cancer to neoadjuvant chemotherapy, MRI-based combined radiomics models are being tested. Zhang et al. showed that, when combined with the clinical T stage, the combined radiomics model of Radscore_T2WI, Radscore_DWI, and Radscore_ADC kept the prominent characteristics of each model and provided the highest AUC. However, the variation of the MRI machines and lesion selection may pose a problem that needs to be overcome.

Another ongoing debate is around what, if any, role diabetes plays in the prevalence and prognosis of UC. Gao et al. conducted a meta-analysis comprising 10 studies and over 11,000 patients to explore the association between diabetes and UTUC prognosis. Interestingly no significant impact of diabetes on survival outcomes was noted, although an increase in intravesical recurrence was seen. The underlying mechanism is proposed to be the overexpression of insulin-like growth factor-1 in UC cells, promoting proliferation and inhibiting apoptosis (4). Diabetes might be a factor to consider when deciding on intravesical adjuvant treatment. However, prospective studies with longer follow-up are needed to address this question.

The final paper in this series, presented by Li et al., explores the role of multimodality treatment for distal ureteral UC. Although radical nephroureterectomy represents the standard for distal ureteral high-grade UC, partial ureterectomy (PU) is an option in selected cases who are at risk for dialysis. This single center retrospective study reported that adjuvant radiotherapy (ART) in stage T3 or grade 3 tumors could significantly improve recurrence-free survival compared with PU alone, and that survival outcomes were comparable between PU+ART and radical nephroureterectomy. This underscores the importance of adjuvant therapies in locally advanced UTUC. As we are in the era of adjuvant systemic therapies for UTUC, a prospective trial to compare ART and adjuvant chemotherapy in this highly selected population would be challenging (5).

## Conclusions

The studies covered here tackle different aspects of research but all aim to bring us closer to improved treatments and outcomes for urothelial carcinoma. Although they present promising results, it is clear that much remains to be done to validate this early work and move us ahead in tackling this common and deadly disease.

## Author contributions

All authors listed have made a substantial, direct, and intellectual contribution to the work and approved it for publication.
